# Gaining Insights Into the Estimation of the Circadian Rhythms of Social Activity in Older Adults From Their Telephone Call Activity With Statistical Learning: Observational Study

**DOI:** 10.2196/22339

**Published:** 2021-01-08

**Authors:** Timothée Aubourg, Jacques Demongeot, Nicolas Vuillerme

**Affiliations:** 1 Orange Labs Meylan France; 2 Univ. Grenoble Alpes AGEIS Grenoble France; 3 LabCom Telecom4Health Univ. Grenoble Alpes & Orange Labs Grenoble France; 4 Institut Universitaire de France Paris France

**Keywords:** circadian rhythms, phone call detail records, older population, statistics, machine learning

## Abstract

**Background:**

Understanding the social mechanisms of the circadian rhythms of activity represents a major issue in better managing the mechanisms of age-related diseases occurring over time in the elderly population. The automated analysis of call detail records (CDRs) provided by modern phone technologies can help meet such an objective. At this stage, however, whether and how the circadian rhythms of telephone call activity can be automatically and properly modeled in the elderly population remains to be established.

**Objective:**

Our goal for this study is to address whether and how the circadian rhythms of social activity observed through telephone calls could be automatically modeled in older adults.

**Methods:**

We analyzed a 12-month data set of outgoing telephone CDRs of 26 adults older than 65 years of age. We designed a statistical learning modeling approach adapted for exploratory analysis. First, Gaussian mixture models (GMMs) were calculated to automatically model each participant’s circadian rhythm of telephone call activity. Second, k-means clustering was used for grouping participants into distinct groups depending on the characteristics of their personal GMMs.

**Results:**

The results showed the existence of specific structures of telephone call activity in the daily social activity of older adults. At the individual level, GMMs allowed the identification of personal habits, such as morningness-eveningness for making calls. At the population level, k-means clustering allowed the structuring of these individual habits into specific morningness or eveningness clusters.

**Conclusions:**

These findings support the potential of phone technologies and statistical learning approaches to automatically provide personalized and precise information on the social rhythms of telephone call activity of older individuals. Futures studies could integrate such digital insights with other sources of data to complete assessments of the circadian rhythms of activity in elderly populations.

## Introduction

### Background

Circadian rhythms are endogenous processes that regulate the individual’s activity over a 24-hour cycle. They are recognized to play a crucial role in nearly all biological and social aspects of the individual’s life, as it is evidenced in his or her brain activity [1], rest activity [2], body temperature fluctuation [3], and rhythms of social interactions [4]. Such rhythms of activity, maintained over time, permit the individual to efficiently organize his or her physical and social activities at a daily scale. Such organization has played a significant role in the past from an evolutionary perspective for ensuring the survival of the human species [5]. Nowadays, circadian rhythms continue to be essential for our lives, giving us the ability to ensure a certain stability in the way we routinely interact with ourselves and with each other.

Furthermore, it is recognized that the circadian rhythms of activity significantly change with age (eg, see Hood and Amir, Steponenaite et al, Zhao et al, and Duffy et al [6-9] for recent reviews). At the endogenous level, the aging process may contribute to the occurrence of biological dysregulations that alter the internal clock of an older individual [10,11]. These changes may have potential adverse consequences on his or her body functioning (eg, see Hood and Amir [6] and Leng et al [12] for recent reviews), such as balance control alteration [13], sleep cycle disruptions [2,14], neurodegeneration [14], or cardiovascular complications [15]. Thus, with regard to our traditional health care system, it seems important for the health practitioner to assess the circadian rhythms of activity of his or her older patients in order to understand, and then to properly manage, the mechanisms of health issues that may occur in their life span.

Over the past decades, the assessment of the circadian rhythms of activity in humans has been addressed at the biological and physical levels through, respectively, the field of chronobiology (eg, see Cornelissen and Otsuka [16] and Otsuka et al [17] for two recent reviews) and that of actigraphy (eg, see Tazawa et al [18] and Schwab et al [19] for two recent reviews). On the whole, both of these approaches—chronobiology and actigraphy—have demonstrated the relevance of combining the use of wearable sensors with the implementation of statistical learning algorithms for automatically measuring the biological and gross motor activities of the individual at a daily scale. From a clinical perspective, these approaches may permit the detection in real time of the occurrence of daily risky situations that may affect the older individual’s life, such as sedentary behavior [20], nocturnal activity [21], fall occurrences [22], or myocardial infarction [23]. Taken together, these approaches provide innovative and relevant ways to diagnose and manage the older adult’s health status over time.

At this point, however, it is also important to mention that the daily rhythms of activity that are of a social nature are evidently not considered by such approaches as relying on biological and physical sources of data. At the clinical level, this issue is of significant importance given that biological circadian dysregulations can be associated with external social factors [24], such as inconsistent mealtime [25], shift work [26], or social isolation [27]. These social disruptions, maintained over time, may lead to a misalignment between biological and social rhythms, so-called “social jet lag” [28]. In the older adult, this misalignment can act as a retroactive feedback mechanism that contributes to the occurrence or worsening of particular symptoms or comorbidity factors, such as fatigue [2,29] and signs of anxiety and depression [30], that are involved in various age-related diseases [12,13,31].

### Prior Work

In the field of health care monitoring for older adults, traditional approaches such as clinical questionnaires propose subjective, timely solutions for measuring and characterizing the circadian rhythms of social activity of the individual. Despite their widespread use in practice, this type of approach introduces limitations into their use for both clinical and research applications. One reason is related to the fact that the administration of questionnaires is time-consuming and requires the active participation of the patient for filling in a given questionnaire. These two constraints cannot be easily satisfied regarding specific vulnerable populations that are potentially not able to make such an effort because of their physical or psychological conditions. A second reason is because of the temporal scope of their results. Since these questionnaires are filled out at a set point in time, it is not possible to infer the general daily rhythms of the individual over a long period of time. A third reason is because of their subjective nature. Subjective responses that are provided for measuring objective temporal phenomena, such as the circadian rhythms of social activity, makes it difficult to ensure the precision of the questionnaires’ results.

Modern technologies traditionally considered as everyday tools have the potential to get around these limits [32-34]. In particular, it has been recently demonstrated that the telephone may have the ability to passively, objectively, and continuously provide estimation of social rhythms through the analysis of its generated data over time [32,35-37]. Notably, two recent descriptive studies [32,38] have analyzed an 18-month data set of call detail records (CDRs) of 24 young adults. The results of these descriptive studies suggested that young participants seemed to hold their own personal circadian patterns for communicating with their social network. More importantly, these circadian patterns were found to persist over time. More recently, such a descriptive analysis was successfully reproduced, and got the same results, with an older population [39]. To the best of our knowledge, these preliminary results were the first ones that specifically concerned the circadian rhythms of telephone call activity of older adults.

Despite this encouraging pioneering work, key limitations remain to be addressed. In particular, regarding older adults, substantial efforts remain in order to improve the descriptive methodology of the recently completed studies in order to properly model the circadian rhythms of telephone call activity. From a health care monitoring perspective, such efforts are crucial for extracting relevant information from the circadian rhythms of telephone call activity that can be used for completing traditional subjective questionnaires.

Thus, this paper is a secondary analysis of recent analyses [39,40] and is specifically designed to address whether and how the circadian rhythms of social activity observed in the telephone calls of older adults could be modeled. To this end, we analyzed a 12-month data set of outgoing telephone CDRs of 26 adults older than 65 years of age. We designed a statistical learning approach adapted for exploratory analysis. First, Gaussian mixture models (GMMs) were calculated to automatically model each participant’s circadian rhythm of telephone call activity. Then, k-means clustering was used for grouping participants into distinct groups depending on the personal characteristics of their GMM curves. The results, the significance, and the limitations of this study are discussed, and perspectives of future research are proposed.

## Methods

Data collection, volunteer recruitment, and data preprocessing followed the general principles set in previous investigations of this secondary analysis [39-43] and remain unchanged for consistency.

### Data Collection and Volunteer Recruitment

Our data set includes 12 months of outgoing CDRs for 26 volunteers (20 women [77%] and 6 men [23%]; mean age 84 years, SD 4; age range 71-91 years). CDRs provided by the local communication service provider were collected from the personal telephones of the volunteers. Each CDR contained the date, hour, source ID, recipient ID, direction, and duration (seconds) of the call. In addition, volunteers who had several telephones registered with their communication service provider (eg, one or more landlines and/or one or more mobile phones) provided CDRs for all their telephones. Finally, only outgoing calls were considered in this study considering ingoing not resulting from a voluntary activity of the participants. Note that the telephone owners and the telephone contacts remained anonymous. This study and the corresponding experimental protocols were approved by the French Commission for Data Protection and Liberties (Correspondant Informatique et Libertés [CIL] Register France Telecom 2011 n°44) at the time the data collection phase of this project was originally initiated. All methods were performed in accordance with the Commission’s regulations, written informed consent was obtained from all participants prior to data collection, and anonymization of participants’ data was applied to ensure privacy requirements.

### Data Preprocessing

Participants did not all enroll in the survey at the same time, so the dates of inclusion varied. Thus, the CDR data set was filtered to select the time interval of when the greatest number of volunteers were actively participating in the study. The CDR data set was then preprocessed by applying the method described by Saramäki et al [38], namely, by selecting only those participants who used their telephones throughout the entire 12-month observation period, which gave a sample of 21 individuals (see Table 1 for details).

**Table 1:** Data set structure of call detail records before and after preprocessing.

Participant characteristics		Before preprocessing (N=26)	After preprocessing (N=21)	
**Number of participants, n (%)**				
	Female	20 (77)		16 (76)
	Male	6 (23)		5 (24)
**Age (years)**				
	Range	71-91		71-91
	Mean (SD)	84 (4)		83 (4)
**Number of calls, n**				
	Total outgoing calls	19,198		18,338
	Calls by individual, 1st quartile	285		481
	Calls by individual, median	590		710
	Calls by individual, 3rd quartile	944		1096

### Data Analysis

#### Overview

To identify social interaction patterns in older adults from their CDRs at a daily scale, we developed a statistical learning approach that is divided into two main processes consisting of the following:

1. Modeling daily rhythms of social interactions in older adults from their CDRs by using GMMs [44].
2. Calculating clusters of morningness-eveningness categories stemming from these modeling results by using k-means clustering [45].

**Figure 1:**
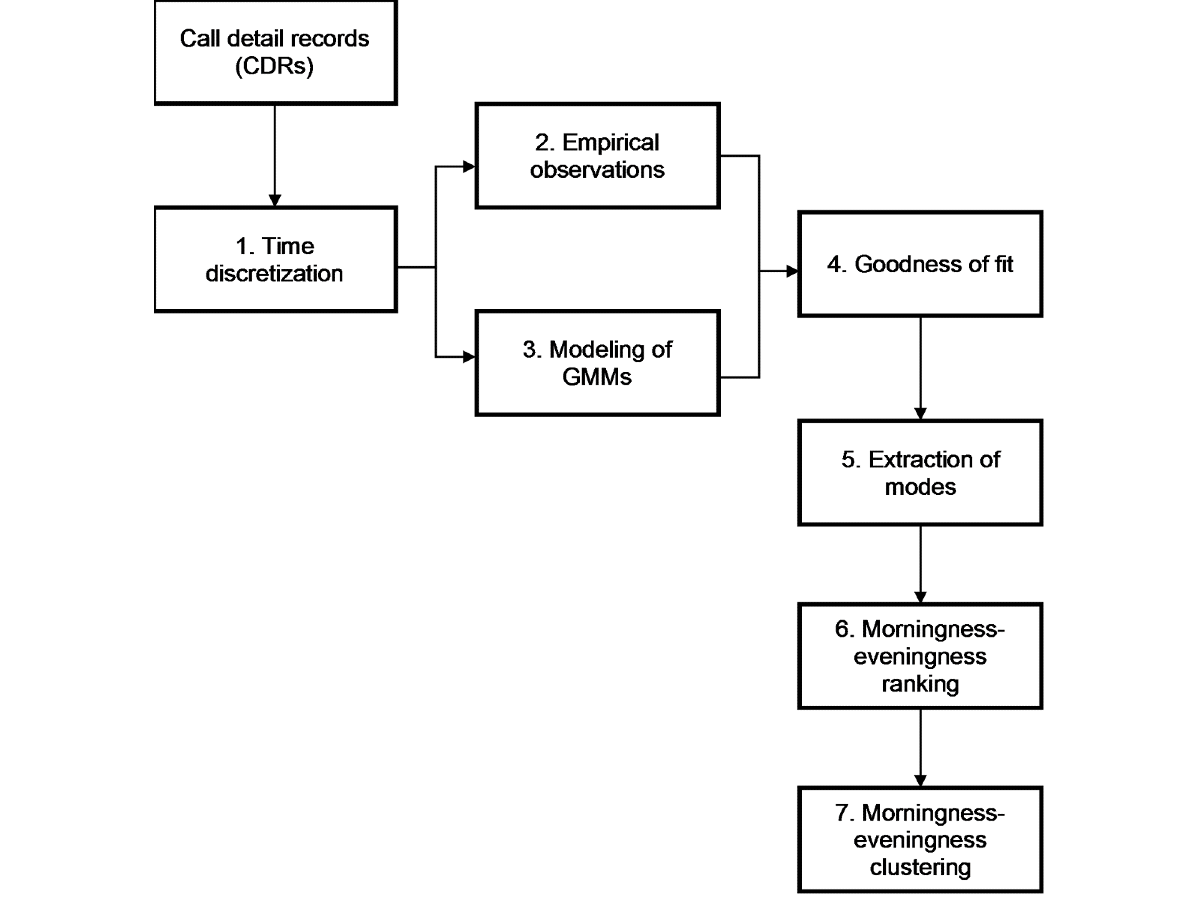
Data analysis pipeline. Step 1 corresponds to data preprocessing, Steps 2-4 correspond to circadian rhythm modeling, and Steps 5-7 correspond to social morningness-eveningness clustering. GMM: Gaussian mixture model.

These processes were chained together throughout our general data analysis pipeline as discussed in the following seven steps (see Figure 1 for an overview).

#### Step 1: Time Discretization

To focus on circadian rhythms of phone call activity, we first discretized the time dimension of CDRs into one unique day of 24 1-hour slots corresponding to the time of day, as previously done in the literature [32,36,39,40,46].

#### Step 2: Empirical Calculation

From this coarse-grained data set, the empirical circadian rhythm of phone call activity was then obtained by calculating, for each older individual inside of each 1-hour time slot, his or her corresponding frequency of phone calls, as in several studies [32,36,39,40,46]. Formally, if we let N be the number of older participants (N=21), and we let *n_i_*(*t*) be the number of phone calls of one individual *i*, with *i* ranging from 1 to N, at a time *t*, with *t* ranging from 0 to 23, then the frequency of phone calls for each individual at a time *t* is defined as follows:


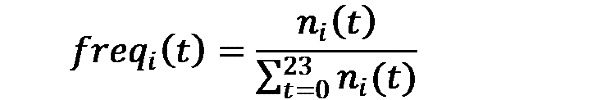


#### Step 3: Modeling of GMMs

In parallel with Step 2, a statistical modeling approach caught the distinct periods of phone call activity across the day by calculating, for each older individual, his or her corresponding GMMs from his or her coarse-grained CDRs. In short, this mixture consisted of synthesizing the older individual’s circadian rhythm of phone call activity by means of distinct curve components. Since these components stem from the fundamental and well-known Gaussian model, their mean and variance characteristic parameters can be easily calculated and used to further characterize the individual’s phone call daily activity. In accordance with the existing literature on the subject [4,47-49], we assumed the existence of two broad periods of activity corresponding to morningness and eveningness in humans, following the diurnal principle, which can be, however, shifted in certain cases toward a potential nocturnal period of activity.

#### Step 4: Goodness-of-Fit Estimation

The goodness of fit of the estimated GMM with respect to empirical data was assessed for each older individual by using a Kolmogorov-Smirnov comparison test under the null hypothesis H_0_—the estimated GMM is similar to the empirical distribution of phone calls across the day—with a *P* value set at .05. Hence, a *P* value greater than .05 represents a significant good fit. This step permits the measurement of the quality of the estimated model with respect to the individual’s empirical observations.

#### Step 5: Extraction of Modes

Next, to focus on the individual’s chronotype, the mean parameter of each Gaussian component, which corresponds to an hourly peak of phone call activity, was extracted for each older individual. The duration of each period of activity was also considered by extracting the variance of each period.

#### Step 6: Morningness-Eveningness Ranking

Since modes were extracted from distinct Gaussian components whose intensities may be different, the individual’s morningness-eveningness characteristic was obtained by assigning each older individual to his or her most representative Gaussian component. To this end, the estimated mixing proportion of each Gaussian component was calculated from an expectation minimization approach for each older adult. Next, the individual’s modes were ranked together according to these proportions to determine the time of day when the older individual was the most socially active.

#### Step 7. Morningness-Eveningness Clustering

Eventually, older adults were categorized into distinct groups according to their ranked modes that were calculated above in Step 6. To this end, each older individual was assigned to a point in a 2-dimensional space that corresponded to his or her two periods of phone call activity. We then used k-means clustering to calculate groups of older individuals from the coordinates of their representative points.

## Results

### Modeling Circadian Rhythms of Outgoing Phone Call Activity in Older Adults With GMMs

**Figure 2:**
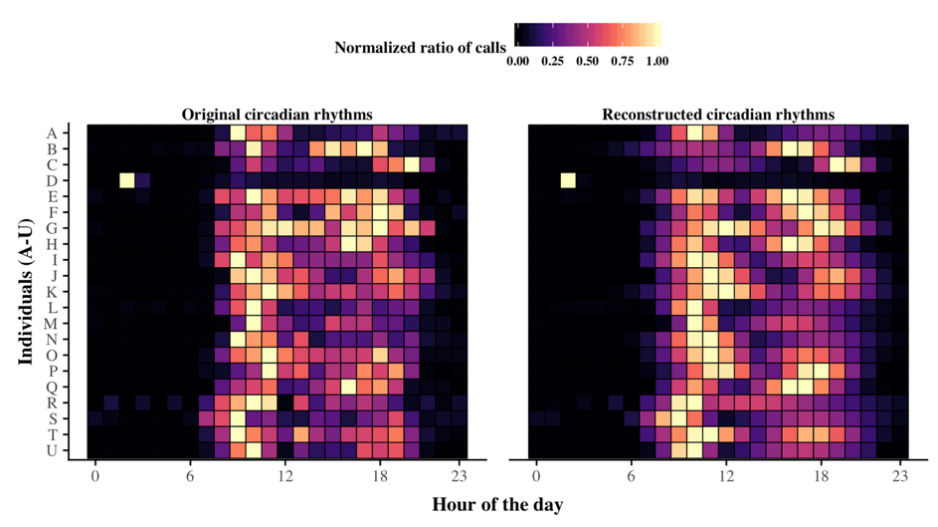
Circadian rhythms of outgoing telephone call activity in older adults and their corresponding Gaussian mixture models (GMMs). These patterns are represented by means of two colored heat maps, where each line corresponds to one older individual and columns correspond to hours of the day. Hence, the outgoing telephone call activity of one individual at a given hour is represented by a colored cell that is all the more bright as the individual increases activity. The left-hand heat map represents the original circadian rhythms of outgoing telephone call activity observed from empirical data, whereas the right-hand heat map represents those modeled by GMMs.

Figure 2 shows, for each older adult, both (1) his or her empirical circadian rhythms of outgoing telephone call activity (see Methods for details) and (2) his or her corresponding GMM (see Methods for details) on two distinct panels. Two observations stand out:

1. On the left-hand panel of Figure 2, the observed empirical circadian rhythms identify the existence of distinct peaks of phone call activity across the day that vary among older individuals. In accordance with previous descriptive results found in Aubourg et al [39], some of them, as with individuals A and I, showed a morning preference for phoning, whereas others, as with individuals C and Q, showed an evening preference. There was also one individual, individual D, who presented a significant shift in his rhythms for phoning, as evidenced by his peak of phone call activity that occurred at night around 2 AM.
2. Comparing empirical data with the models of GMMs for each older individual, we observed on the right-hand panel of Figure 2 the ability of GMMs to highlight periods of high activity and for smoothing periods of weak activity by the smoothing property of the Gaussian curve.

**Figure 3:**
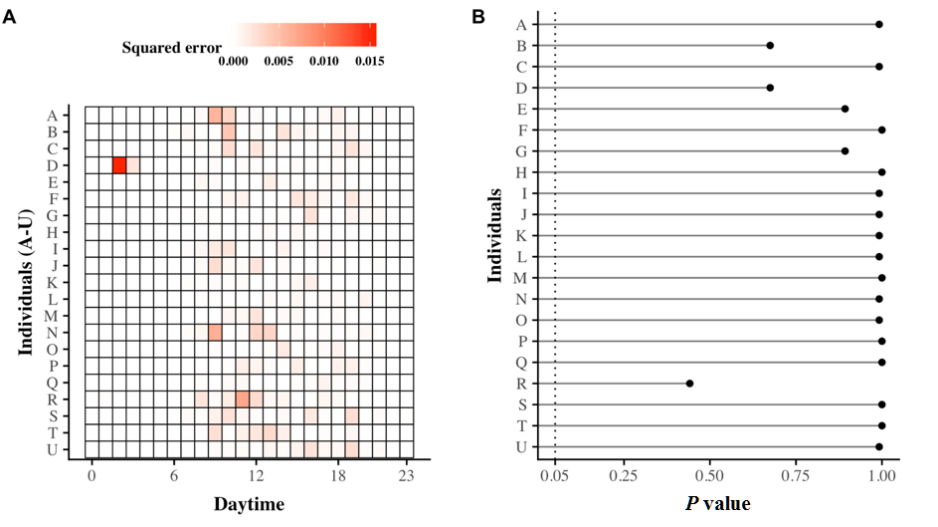
Goodness of fit of Gaussian mixture models (GMMs). Panel A corresponds to a heat map that figures out the squared errors calculated between empirical observations and their corresponding GMMs. Each line corresponds to one older individual, whereas columns correspond to hours of the day. Hence, the squared error between the outgoing telephone call activity and the estimated model at a given hour is represented by a colored cell that is all the more bright as the error increases. Panel B displays the associated goodness of fit by giving *P* values from the Kolmogorov-Smirnov comparison tests. Here, the significance threshold is set at .05 (dashed line).

Furthermore, at a statistical level, Figure 3 provides the goodness of fit of the GMMs. In this figure, we observe that the similarity between empirical observations and GMMs cannot be rejected for each of the participants, with a *P* value greater than .05, based on the Kolmogorov-Smirnov comparison test.

### Social Morningness-Eveningness Is Evidenced in the Telephone Call Activity of Older Adults

**Figure 4:**
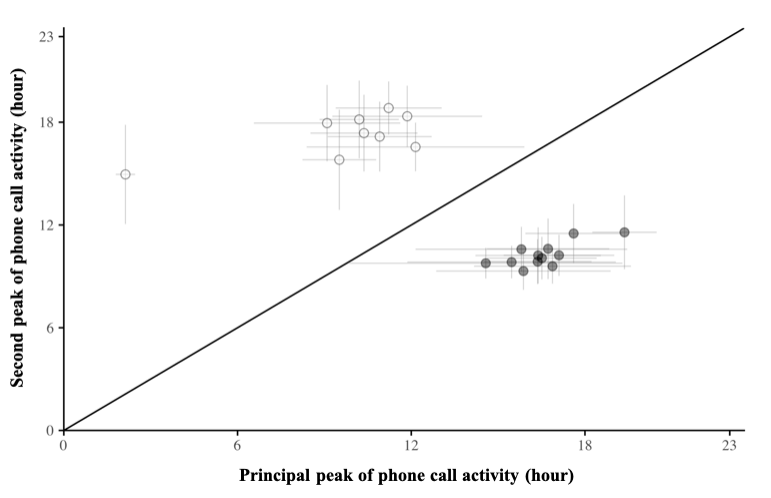
Morningness-eveningness observed in outgoing telephone calls. This figure displays the two broad periods of outgoing telephone call activity. Circles represent peaks of telephone call activity, and each period duration is represented with horizontal and vertical lines. Each circle is colored according to its morningness-eveningness trend given by k-means clustering.

The k-means clustering process identified distinct categories of older individuals based on the characteristics of their daily outgoing telephone call activity. Figure 4 shows the k-means clustering results by displaying the two broad periods of telephone call activity of each older individual. This figure highlights the individual nature of social behavior for making telephone calls over the day. In the same way, it stands out that two broad patterns can be conceived in our data set, namely (1) *morningness* individuals have their principal period of telephone call activity in the morning and their second period later in the afternoon or evening and (2) *eveningness* individuals have the exact opposite behavior. Bipartite clustering confirmed this general trend by explicitly exhibiting both of these clusters. Again, we also have to point out that one older adult belonging to the *morningness* cluster, individual D, had an *extreme* behavior whose expression differed from the others, with his first peak of telephone call activity occurring at around 2 AM.

## Discussion

### Principal Findings

Analysis of daily social interactions by means of CDRs is now recognized as a relevant approach for modeling human activity in an objective, unobtrusive way, which offers new opportunities for improving the field of health monitoring [50,51]. However, in the elderly population, more work is required to confirm the ability of such an approach to model relevant patterns of social interactions at a daily scale. This study is specifically designed to address this issue. To this end, we used a 12-month data set of outgoing CDRs of 26 adults older than 65 years of age. On the whole, our results allowed us to automatically model circadian patterns of outgoing telephone call activity. Interestingly, the results obtained at the individual level suggest the existence of individual hourly preferences for making telephone calls during the day. Despite the evident individual nature of such preferences shown by GMMs, the results also suggest the possibility for assembling these individual observations into broader categories using k-means clustering. In particular, these categories of phone call activity were of two broad types, namely (1) *morningness* older adults that had their main period of telephone call activity in the morning with their second one occurring later, in the afternoon or evening, and (2) *eveningness* older adults that had the exact opposite behavior.

With regard to the field of social physics applied to CDR data sets [52], these results corroborate recent findings about the existence of daily patterns in human social interactions over the telephone, which were exhibited at the population level [53]. Monsivais et al [53] analyzed a 12-month CDR data set containing more than 3 billion calls between 50 million unique identifiers. By aggregating data at the population level, the authors described the existence of an overall bimodality in outgoing telephone call activity that occurred at noon and during the evening periods of around 8 hours. Similarly, they also showed the existence of two periods of around 4 hours of low calling activity occurring (1) at night and (2) after lunch time. Such results permitted the authors to infer the existence of rhythms for representing the resting times of the population in order to study the impact of seasonal and geographical elements on that population. However, the focus on aggregate data from that study [53] does not allow the authors to reach a conclusion regarding the individual rhythms of the studied population because of ecological fallacy, nor within specific populations such as the aging one.

In a complementary way and for the first time to the best of our knowledge, this paper provides evidence at the individual level of the ability of statistical learning approaches to model similar results as those obtained by Monsivais et al [53] within a small cohort of older adults.

Furthermore, two singularities also stand out regarding our results in comparison with those obtained by Monsivais et al [53]. On the one hand, the bimodality exhibited in our older population seemed to appear earlier during the day at midmorning around 10 AM and in the evening around 6 PM. Knowing that the morning transition tends to appear with age, it is plausible that this observed shift, in comparison with the general behavior exhibited in the study by Monsivais et al [53], may reflect such a transitive state and could consequently be a representative element of the aging population [47]. On the other hand, our results also show the existence of nocturnal patterns of telephone call activity within our aging population that could occur in some older individuals. It is plausible that such nocturnal activity could reflect particular disruptions such as sleep disturbances, which are known to occur with age [54].

Hence, the analysis of social interactions in older adults by means of their CDRs does provide evidence about the existence of both social and structural insights about the older individual’s daily social life. From a clinical perspective, such insights offer a relevant opportunity to consider modern phone technologies as a new kind of social activity sensor whose captured data can be automatically modeled to identify the fundamental social dynamics in the older individual’s life. Social dynamics regarding telephone use could notably be harnessed to detect potential disruptions, such as nocturnal phase shifts or chronotype alterations, or to prevent the occurrence of particular health issues, such as sleep disorders, being presented to the clinician in the form of triggers.

Furthermore, since these pieces of information about social interactions do present the advantage of being collected in a passive, objective, and continuous way, they could be harnessed in complement with other timely subjective methods, such as subjective questionnaires [4,49], but also with innovative digital technological approaches such as *actigraphy*, which already exists for monitoring physical rhythms of the individual [55,56].

### Study Limitations and Perspectives

A number of caveats and limitations have to be taken into account. Since this study’s analysis was based on a relatively small sample of 26 older individuals, any straightforward generalization of our results to the overall older population should be avoided. Given the sample size of this study, at this stage the interest of our exploratory work is not in providing general knowledge on older adults’ social activity on the telephone but, rather, in the ability to gain new insights that deserve to be included for future big data studies on improved data sets. Consequently, whether and how similar results could be observed under different conditions using different sets of data or by implementing alternative statistical methods should naturally be investigated. This could imply working on larger data sets but also, more broadly, leading analyses on distinct populations having their own social characteristics, such as individuals from particular social classes or from different cultures. Given the proportion of women represented in the analyzed data set (76%), whether and how similar works can be replicated with success within populations that have different proportions of male and female participants deserve to be addressed. Furthermore, it could be interesting to focus on specific cohorts suffering from age-related diseases, such as Alzheimer disease [31], Parkinson disease [13], and depression [30,57]. These supplementary works are all the more important insofar as, to the best of our knowledge, this study is the first one that investigates daily patterns of social interactions in an aging population specifically by combining CDR analysis with a statistical learning approach.

Among future perspectives, an essential step that remains to be investigated is whether, and to what extent, the results presented here could be harnessed to enhance already-existing digital frameworks in health research. Such an investigation could consist of developing an adequate complementary framework based on both judiciously used, statistical learning methods and behavioral theory to permit a better understanding of the digital profile of the older individual. In the field of health care monitoring, we do believe that such an investigation could represent a relevant approach for enhancing the interpretability of results and the efficiency of modeling approaches relying on telephone call activity.

### Conclusions

Findings from this study support the potential of phone technologies and statistical learning approaches for automatically providing personalized and precise insights on the social rhythms of telephone call activity of older individuals. Such insights can highlight particular individual habits related to being active on the telephone according to the time of day. Furthermore, these individual pieces of information can be automatically analyzed by statistical learning approaches at the group level in order to provide more general information on the observed cohort, such as subtrends for clusters of older individuals, for instance, who are more socially active on the telephone in the morning or in the evening. Futures studies could integrate such digital insights with other sources of data to complete the assessment of the circadian rhythms of activity in the elderly population. In particular, big data studies should be implemented with large cohorts in order to investigate to what extent our study’s results that were obtained from our sample of older participants at the group level can be transformed into broad actionable knowledge for the older population.

## Abbreviations

CDR: call detail record
CIL: Correspondant Informatique et Libertés
GMM: Gaussian mixture model
